# The mRNA-Binding Protein RBM3 Regulates Activity Patterns and Local Synaptic Translation in Cultured Hippocampal Neurons

**DOI:** 10.1523/JNEUROSCI.0921-20.2020

**Published:** 2021-02-10

**Authors:** Sinem M. Sertel, Malena S. von Elling-Tammen, Silvio O. Rizzoli

**Affiliations:** ^1^Institute for Neuro- and Sensory Physiology, University Medical Center Göttingen, Göttingen, 37073, Germany; ^2^Cluster of Excellence “Multiscale Bioimaging: from Molecular Machines to Networks of Excitable Cells” (MBExC), University of Göttingen, Göttingen 37073, Germany

**Keywords:** circadian, local translation, primary hippocampal culture, RBM3, RNA-binding protein, synaptic transmission

## Abstract

The activity and the metabolism of the brain change rhythmically during the day/night cycle. Such rhythmicity is also observed in cultured neurons from the suprachiasmatic nucleus, which is a critical center in rhythm maintenance. However, this issue has not been extensively studied in cultures from areas less involved in timekeeping, as the hippocampus. Using neurons cultured from the hippocampi of newborn rats (both male and female), we observed significant time-dependent changes in global activity, in synaptic vesicle dynamics, in synapse size, and in synaptic mRNA amounts. A transcriptome analysis of the neurons, performed at different times over 24 h, revealed significant changes only for RNA-binding motif 3 (Rbm3). RBM3 amounts changed, especially in synapses. RBM3 knockdown altered synaptic vesicle dynamics and changed the neuronal activity patterns. This procedure also altered local translation in synapses, albeit it left the global cellular translation unaffected. We conclude that hippocampal cultured neurons can exhibit strong changes in their activity levels over 24 h, in an RBM3-dependent fashion.

**SIGNIFICANCE STATEMENT** This work is important in several ways. First, the discovery of relatively regular activity patterns in hippocampal cultures implies that future studies using this common model will need to take the time parameter into account, to avoid misinterpretation. Second, our work links these changes in activity strongly to RBM3, in a fashion that is independent of the canonical clock mechanisms, which is a very surprising observation. Third, we describe here probably the first molecule (RBM3) whose manipulation affects translation specifically in synapses, and not at the whole-cell level. This is a key finding for the rapidly growing field of local synaptic translation.

## Introduction

Maintaining a synchronous pattern of day and night activity is critical for the function of all of the tissues of a mammalian organism. This is ensured by several well-established mechanisms, the first of which is the rhythmic expression of molecular clock genes in every cell throughout the day/night (Partch et al., 2014). These genes control the timing of many biological functions, such as glucose metabolism and electrical activity (Dibner et al., 2010). A second fundamental mechanism is provided by the function of the suprachiasmatic nucleus (SCN), a central pacemaker of the hypothalamus, which is in charge of the molecular clock synchronization among the cells of the animal (Welsh et al., 2010). The SCN achieves this by encoding time information in its spontaneous firing rate (low during the night, and high during the day) (Colwell, 2011), and by communicating this to other brain regions and tissues through synaptic projections and hormones (Buijs et al., 2006).

The rhythmic expression of clock genes in the SCN controls the expression and function of ion channels as the BK channels (large-conductance calcium-activated potassium channels) or L-type voltage-gated calcium channels (Colwell, 2011). The function of these proteins induces oscillations in the resting membrane potential (Pennartz et al., 2002; Kononenko et al., 2008), thereby changing the firing rates, which in turn ensures the rhythmic firing activity of the SCN, which has been demonstrated even in dispersed cultures (Green and Gillette, 1982; Herzog et al., 1998). The rhythmic firing is resistant to disturbances in the light-dark cycle (Kuhlman and McMahon, 2004; Nakamura et al., 2011), and it persists in SCN cultures that are not subjected to day/night light or temperature changes. However, clock gene expression alone is not sufficient to maintain the synchronized firing of SCN neurons in the long-term. In culture, they slowly become desynchronized, with every cell eventually assuming its own individual firing pattern that oscillates throughout the day and night cycle (Welsh et al., 1995). The desynchronization is accelerated by blocking network activity, suggesting that neuronal communication is important in maintaining the rhythm synchronicity for long time intervals (Honma et al., 2000; Yamaguchi et al., 2003).

The observation of rhythmic activity in dispersed SCN cultures prompted research also in other cell types. Fibroblast cell lines were found to exhibit molecular clock rhythmicity, albeit they lose cell synchronicity rapidly (Nagoshi et al., 2004), unless they are resynchronized by regular changes in temperature (Brown et al., 2002) or culture media (Balsalobre et al., 1998). However, many brain areas have been little investigated in relation to rhythmic activity (Paul et al., 2020). A prominent example is the hippocampus, which is involved in learning and memory, two processes that are strongly regulated by the circadian clock (Gerstner and Yin, 2010). Hippocampal activity *in vivo* oscillates throughout the day and night cycle (Munn and Bilkey, 2012), and its ability to respond to plasticity-inducing stimuli is also dependent on the time of day/night (Harris and Teyler, 1983). This demonstrates that the hippocampus function is governed by the 24 h cycle but leaves open the question of whether this is exclusively because of the general rhythmicity induced by the SCN, or whether this is a fundamental hallmark of the hippocampal neuron, which would persist in dissociated cultures.

To solve this question, we turned to the rat hippocampal culture. Surprisingly, we found that the culture activity exhibited significant oscillations throughout 24 h, which were accompanied by substantial changes in presynaptic activity and synapse size. In addition, we found that the abundance of RNA-binding motif 3 (RBM3), a cold-shock protein (Danno et al., 1997, 2000) that is known to promote translation (Dresios et al., 2005), also oscillates throughout 24 h, especially in synapses. Its knockdown changed the activity pattern of the neurons, as well as synapse activity and size, possibly through effects on local translation. Overall, these data suggest that hippocampal cultures exhibit endogenous changes in activity levels across 24 h, and that these patterns are under the control of RBM3.

## Materials and Methods

### 

#### 

##### Hippocampal cultures

Primary disassociated hippocampal cultures were prepared from newborn rats (Banker and Cowan, 1977). The hippocampi were dissected from rat brains, using animals of both sexes, with a general female to male ratio of 1:1. They were washed with Hanks balanced salt solution (Thermo Fisher Scientific). Later on, hippocampi were kept in the enzyme solution (1.6 mm cysteine, 100 mm CaCl_2_, 50 mm EDTA, and 25 units papain in 10 ml DMEM) for 1 h. To inactivate the enzyme solution, 5 ml DMEM (Thermo Fisher Scientific) that contains 10% FCS, 0.5% albumin, and 0.5% trypsin inhibitor was added and incubated for 15 min. Cells were further separated by mechanical disruption and were seeded on poly L-lysine (Sigma Millipore) coated circular coverslips (1.8 cm in diameter) with a density of 80,000 cells per coverslip. The neurons were kept in plating medium (3.3 mm glucose, 2 mm glutamine, and 10% horse serum in DMEM) for 1-2 h at 37°C. Afterward, the medium was exchanged to Neurobasal-A medium (with B27 supplement, 1% GlutaMax, and 0.2% penicillin/streptomycin mixture). The cultures were maintained at 37°C and 5% CO_2_ for ∼20 d.

##### Calcium imaging

Neurons were transduced with 3 µl of NeuroBurst Orange Lentivirus (Sartorius) at DIV10, and kept in the incubator for 9 additional days. For imaging, the coverslips were placed into imaging chamber and imaged with and inverted Nikon Ti eclipse epifluorescence microscope equipped with a 20× Plan Apo (Nikon) objective, an HBO-100W lamp, an IXON X3897 Andor camera, and a cage-incubator (Okolab). The temperature was set at 37°C and the atmosphere with 5% CO_2_ throughout the imaging session. For long-term recordings, neurons were plated in a glass-bottom 24-well plate (Cellvis) and imaged directly from the plate.

##### Promoter reporter imaging

The plasmid for the promoter reporter imaging was synthesized by GenScript, using pUC57 as a backbone. The promoter was selected as the sequence from 500 nucleotides in the upstream until 50 nucleotides in the downstream of the BMAL1 gene from *Rattus novergicus*. The plasmid expressed EGFP under the control of this promoter. The EGFP was destabilized by adding to its C terminus the residues 422-461 of mouse ornithine decarboxylase, which provides a 2 h half-life time for the molecule (Li et al., 1998). Neurons were transfected with Lipofectamine 2000 (Thermo Fisher Scientific) at DIV5, according to the manufacturer's instructions.

##### Immunostaining

Neurons were washed with the Tyrode's buffer (124 mm NaCl, 5 mm KCl, 2 mm CaCl_2_, 1 mm MgCl_2_, 30 mm D-glucose, and 25 mm HEPES) and then fixed with 4% PFA (Sigma Millipore) for 30 min at room temperature. Later on, cells were incubated in the quenching solution (100 mm NH_4_Cl in PBS) for 30 min at room temperature. Subsequently, neurons were washed with permeabilization solution (3% BSA, 0.01% Triton-X-100 in PBS) 3 times for 5 min on a shaker. Permeabilized neurons were incubated for 1 h, with 0.2% of the primary antibody in the permeabilization solution. Then, they were washed again with permeabilization solution 3 times for 5 min on a shaker. Neurons were incubated for 1 h with 0.5% of the secondary antibody in permeabilization solution. Later on, they were washed with high-salt PBS (supplemented with 0.38 m NaCl) solution 3 times for 5 min on a shaker and 2 times for 5 min with PBS. Last, coverslips were mounted in 8 µl Mowiol (Merck Millipore) and stored at 4°C. Unless otherwise specified, imaging was performed with IX83 inverted Olympus confocal microscope (Abberior) that is equipped with a 100× super-apochromat and coverslip-corrected oil objective (Olympus). The analysis was performed on MATLAB (The MathWorks) and plotted with GraphPad. The Syph (101004) and Homer1 (160011) antibodies were purchased from Synaptic Systems, and the RBM3 antibody (ab134946) was purchased from Abcam.

##### Synaptotagmin1 uptake assay

In order to study the synaptic vesicle usage, we took advantage of live staining with an antibody targeting the luminal domain of Synaptotagmin1. At DIV21, coverslips with neurons were placed in a new 12-well plate (Greiner Bio-One) with 300 μl of their own Neurobasal-A medium. Neurons were incubated with 2.5 μg/ml Syt1-Atto647N antibody (105311AT1, Synaptic Systems) for 45 min. Afterward, 16.7 nm anti-mouse secondary nanobody (N2002-At542-S, Nanotag) conjugated to Atto542 was added into the medium and incubated for15 min. Next, the neurons were washed with ice-cold Tyrode's buffer and fixed with 4% PFA. The immunostaining procedure for synaptophysin is described in Immunostaining. In order to see the spontaneous synaptic vesicle fusion, the action potential generation was blocked by adding 5 μm TTX (Tocris Bioscience). In time-series experiments, the uptake assay was performed at different time points of the day and night. For the knocked down conditions, the uptake assay did not have secondary nanobody incubation. Instead, Syt1-Atto647N antibody was incubated for either 15 min or 1 h. An inverted Nikon Ti eclipse epifluorescence microscope (Nikon), which has a 20× Plan Apo (Nikon) objective, an HBO-100W lamp, and an IXON X3897 Andor camera, was used for imaging, and the images were analyzed using MATLAB (The MathWorks).

##### Puromycin assay

Puromycin (ant-pr-1, InvivoGen) is an antibiotic that interferes with mammalian translation and incorporates itself into the polypeptide chain. Coverslips were placed in a new 12-well plate with 300 μl of their own Neurobasal-A medium and were incubated with 1 μl of 0.3 mg/ml puromycin for 10 min in the incubator. Later on, they were washed twice with ice-cold Tyrode's buffer and fixed with 4% PFA. As a control, another antibiotic called anisomycin was used. It halts the translation complex and does not allow puromycin to reach the binding site in the ribosome. Control groups were incubated with 0.13 μm anisomycin (A5862, Sigma Millipore) 10 min before puromycin treatment. Later, the immunostainings against synaptophysin, Homer1, and puromycin (MABE343, Merck Millipore) were performed as described in Immunostaining. Puromycin, anisomycin, and puromycin antibody were generous gifts from Prof. Peter Rehling (University Medical Center Göttingen, Göttingen, Germany).

##### Poly(A) staining

Oligo(dT) and oligo(dA) stainings were done as described previously (Chou et al., 2018). Briefly, neurons were fixed and quenched, as stated in Immunostaining. They were fixed one more time with ice-cold absolute methanol for 10 min. Cells were rehydrated first with 70% EtOH and then with 1 m Tris buffer, pH 8, for 10 min. Later on, neurons were washed with hybridization buffer [1 mg/ml yeast tRNA, 0.005% BSA, 10% dextran sulfate, 25% formamide in finalized 2× SSC (0.3 m NaCl, 30 mm trisodium citrate in water)] once and then incubated with 1:1000 of 1 µg/µl 30 nucleotide long either oligo(dT) or oligo(dA) conjugated to Atto647N (Sigma Millipore) for 1 h in hybridization buffer at 37°C. Samples were washed 2 times with 4× SSC and 2 times 2× SSC. The following immunostainings against synaptophysin and Homer1 were performed as specified in Immunostaining.

##### Transcriptomics

The mRNAseq experiments as well as the analysis were performed by Transcriptome and Genome Analysis Laboratory. Samples were sequenced with HiSeq-4000 (Illumina) with 50 bp single-end design. The alignment was performed with STAR 2.5.2a (Dobin et al., 2013), and the assignment of reads to genes was done by using featureCounts 1.5.0 (Liao et al., 2019) with *R. norvegicus* genome assembly rn6 and gene version 91. After the count calculation of each transcript, we used the limma package to find differentially expressed transcripts (Ritchie et al., 2015). GO analysis was performed on the Webgestalt database with Ensembl gene IDs and difference folds between time points (Wang et al., 2017). The result of gene set enrichment analysis reports the pathways with *p* < 0.05 and false discovery rate < 0.05 (Table 1).

**Table 1.** The biological process pathways determined by analyzing difference among the transcriptomes measured at different time points

	Description	Normalized enrichment score
08:00 vs 14:00	Ribonucleoprotein complex subunit organization	−1.92
	RNA splicing	−2
	Ribonucleoprotein complex biogenesis	−2.32
08:00 vs 20:00	Ribonucleoprotein complex biogenesis	−2.23
	Axon development	1.94
	Cell morphogenesis involved in differentiation	1.95
	Dendrite development	1.93
	Synapse organization	2
	Semaphorin-plexin signaling pathway	1.9
	Cell part morphogenesis	1.91
	Synaptic transmission, glutamatergic	1.97
	Regulation of neuron projection development	1.88
	Neuron projection organization	1.86
08:00 vs 02:00	Vesicle-mediated transport in synapse	1.96
	Synaptic vesicle cycle	1.97
	Glutamate receptor signaling pathway	1.97
	Response to ammonium ion	2
	Serotonin receptor signaling pathway	1.93
	Amine transport	2.02
	Regulation of trans-synaptic signaling	1.92
	Response to anesthetic	1.92
	Synaptic transmission, GABAergic	2.03
	Regulation of postsynaptic membrane Neurotransmitter receptor levels	1.9
20:00 vs 14:00	Defense response to other organism	1.99
	Cytokine-mediated signaling pathway	1.98
	Endothelium development	1.97
	Integrin-mediated signaling pathway	1.96
	Leukocyte migration	1.91
	Humoral immune response	1.85
02:00 vs 14:00	Cell adhesion mediated by integrin	2.25
	Cilium organization	2.16
	Smoothened signaling pathway	2.06
	Extracellular structure organization	2.05
	Integrin-mediated signaling pathway	1.96
	Embryonic morphogenesis	1.87
	Skeletal system development	1.87
	Connective tissue development	1.84
	Cardiovascular system development	1.82
	Skin development	1.8
02:00 vs 20:00	Cilium organization	2.38
	Smoothened signaling pathway	2.29
	Amine transport	−2.23
	Synaptic transmission, GABAergic	−2.42
	GABA signaling pathway	−2.16
	Synaptic vesicle cycle	−2.03
	Neurotransmitter transport	−2.03
	Regulation of neurotransmitter levels	−1.99
	Acid secretion	−1.96
	Response to amine	−2

We performed a gene set enrichment analysis by comparing the transcriptomes obtained at different time points, using the Webgestalt database (Wang et al., 2017). The table shows up to 10 nonredundant biological process pathways whose *p* value was < 0.05 and false discovery rate was < 0.05.

##### short-hairpin RNA (shRNA) virus preparation

The sequence for shRNA was prepared with the help of the BLOCK-iT RNAi Designer database (Thermo Fisher Scientific). The shRNA sequence was synthesized by Genscript and placed in the pAAV-U6sgRNA (60958, Addgene) (Swiech et al., 2015). The plasmid of scrambled (Scr) shRNA was a generous gift of the Fornasiero laboratory (Keihani et al., 2019). Adeno-associated virus was produced in human embryonic kidney 293T (DSMZ) with three plasmids that have packaging proteins of recombinant adeno-associated virus, and were described previously (McClure et al., 2011). Human embryonic kidney cells were transfected with Lipofectamine 2000 (Thermo Fisher Scientific) using the manufacturer's instructions. Three days later, transfected cells were harvested and centrifuged. The pellet was resuspended in 1 ml Tyrode's buffer and was exposed to freeze/thaw cycles 3 times in 70% EtOH and dry ice mixture for lysis. After the addition of 1 µl of Nuclease (Thermo Fisher Scientific), the lysate was incubated at 37°C for 30 min and was centrifuged with 1000 × *g* for 5 min. The supernatant was aliquoted and stored at the −80°C freezer. The virus titration was performed by observing the GFP signal from serial dilution on transfected hippocampal cultures. The virus was used on the primary hippocampal culture at DIV15. The RBM3 shRNA sequence is as follows: CACCGCGTCTTCCCGCGCCGCAGTCCGAAGACTGCGGCGCGGGAAGACGCTTTTTTTT. The BMAL1 shRNA sequence is as follows: CACCGCAAACTACAAGCCAACATTTCGAAAAATGTTGGCTTGTAGTTTGCTTTTTTTT.

##### Experimental design and statistical tests

**Figure 1.**
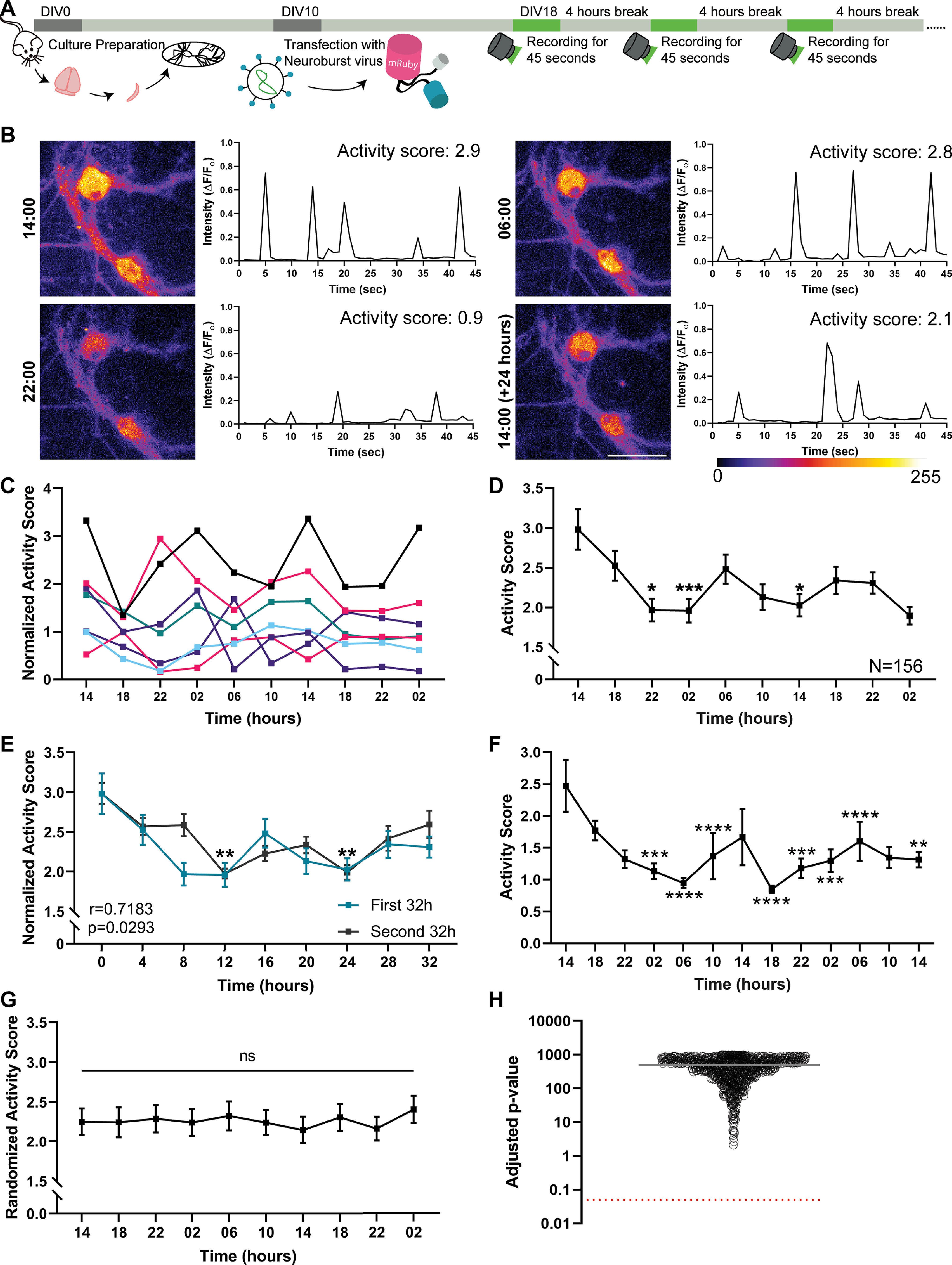
The average neuronal activity in dissociated hippocampal cultures oscillates during the day/night. ***A***, To determine the firing pattern of dissociated hippocampal neurons, we transfected cultured hippocampal neurons with the genetically encoded Ca^2+^ indicator NeuroBurst at DIV10. Starting at DIV18, we imaged neurons every 4 h, relying on a continuous 45 s recording protocol. This provided a sample of activity at the particular time points, while being sufficiently mild to avoid phototoxicity. ***B***, To visualize the overall activity at every time point, we generated summed frames that illustrate the total activity along the 45-s-long videos. The activity in all movie frames, measured in the neuronal cell bodies, is shown in the graphs, in the form of fluorescence normalized to the baseline (ΔF/F_0_). To obtain a single numeric value that represents the activity along the whole movie, we calculated the area under the peaks from these graphs, which we termed “activity score.” Scale bar, 50 μm. ***C***, Seven independent neurons are shown as examples. To enable a simple visual comparison of the traces, they were all normalized to their median activity score. ***D***, To reveal the average firing pattern of the hippocampal cultures, we analyzed 156 neurons, from four independent experiments (with 2-4 different wells measured per experiment). Graph represents their average activity score (±SEM). The statistical significance of changes throughout the experiment was measured by the Friedman test, followed by Dunn's multiple comparisons test. The first time point was the reference for the multiple comparison. *p* = 0.0195 for 14 versus 22. *p* = 0.0002 for 14 versus 02. *p* = 0.0318 for 14 versus 14. *p < 0.05. ***p < 0.001. ***E***, The activity scores across the first 32 h of the recordings (blue) are plotted along with the second 32 h (black). A similar pattern can be observed, which is confirmed by a statistical analysis performed using the Pearson's correlation coefficient (*p* = 0.0293, *r* = 0.7183). Symbols represent time points that exhibited significantly lower activity than for the first time point. **p < 0.01 (Kruskal–Wallis test, followed by Dunn's multiple comparisons test, *p* = 0.0099 for 0 vs 12, and *p* = 0.0043 for 0 vs 24), when the analysis was performed across individual cultures, rather than individual neurons, as in ***D***. ***F***, Average firing pattern of younger, DIV10 cultures (71 neurons from three independent experiments, with 12 different wells measured per experiment). Graph represents the average activity score (±SEM). The statistical significance of changes throughout the experiment was measured by the Friedman test, followed by Dunn's multiple comparisons test. The first time point was the reference for the multiple comparison. *p* = 0.0003877 for 14 versus 02. *p* = 0.0000043428 for 14 versus 06. *p* = 0.000011996 for 14 versus 10. *p* = 0.0000000030882 for 14 versus 18. *p* = 0.0007033 for 14 versus 22. *p* = 0.000244 for 14 versus 02. *p* = 0.00009152 for 14 versus 06. *p* = 0.0024041 for 14 versus 14. **p < 0.01. ***p < 0.001. ****p < 0.0001. ***G***, To confirm that the significant differences observed in the average firing pattern from ***D*** were not because of chance, the activity scores of the neurons were randomized, and the average activity was then investigated by Friedman tests, as in ***D***. An exemplary randomized activity score is plotted as mean ± SEM. ***H***, The randomization procedure was performed 1000 times, and the *p* values that were obtained from the Friedman test were adjusted for the repeated multiple testing. Black circles represent the resulting *p* values. Gray line indicates the mean value. Red dotted line indicates the significance level (0.05).

**Figure 2.**
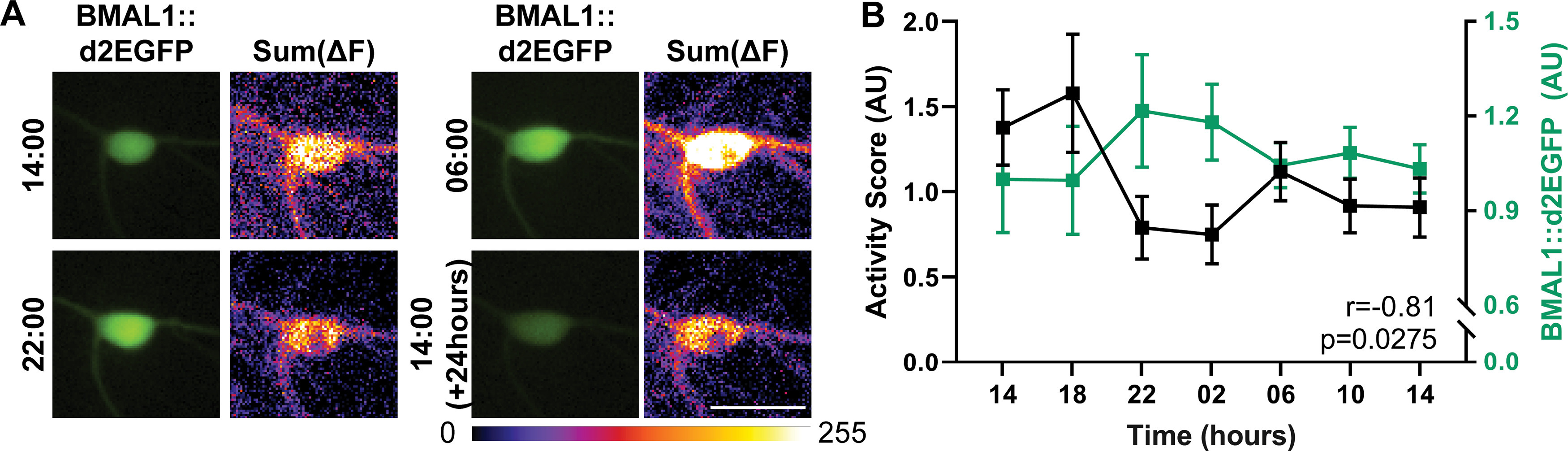
The BMAL1 promoter activity is negatively correlated to the firing pattern in hippocampal culture. ***A***, We analyzed neuronal activity using the genetically encoded Ca^2+^ indicator NeuroBurst, as in Figure 1. The neurons were also transfected with a destabilized EGFP (d2EGFP), under the control of the BMAL1 promoter, which enables us to analyze both BMAL1 promoter activity and neuron activity at the same time. The exemplary images show the d2EGFP fluorescence at different time points, as well as the overall activity at the respective time points, obtained by summing all frames collected in 45-s-long videos, as in Figure 1. Scale bar, 50 μm. ***B***, Graph represents the average activity scores (black) and d2EGFP signals (green), over 24 h. Symbols represent mean ± SEM, from 11 neurons tracked in three independent experiments. The correlation between the two curves is negative, as assessed by the Pearson's correlation test (*r* = −0.81, *p* = 0.0275).

**Figure 3.**
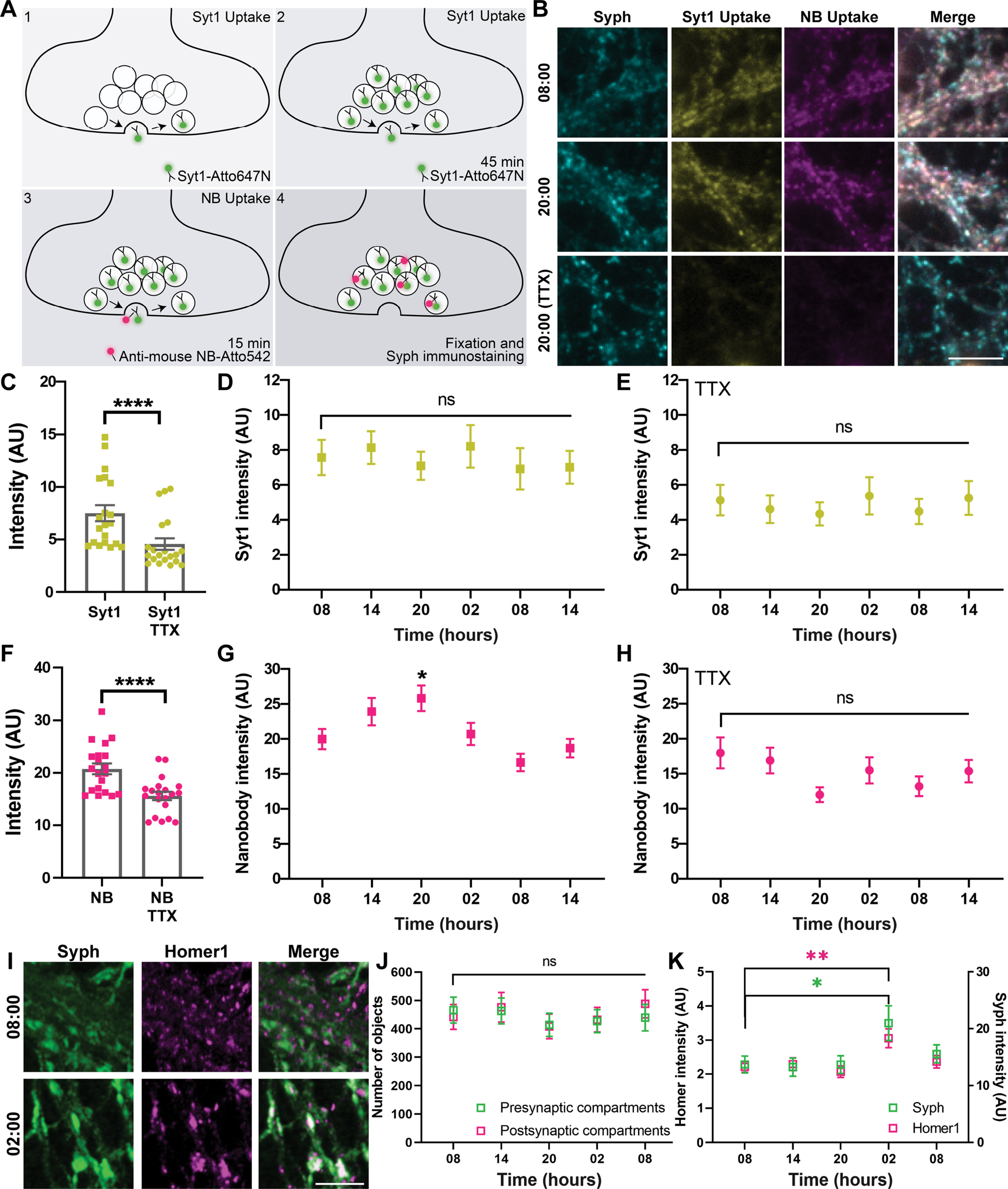
Synaptic vesicle recycling measurements confirm the existence of activity oscillations throughout 24 h. ***A***, To measure the presynaptic activity, we performed a Syt1 uptake assay (Matteoli et al., 1992; Kraszewski et al., 1995) at DIV18, at different times of day and night. To label the recycling vesicles, an Atto647N-conjugated Syt1 antibody was added to the cell culture medium for 45 min (1). The antibody recognizes a luminal (intravesicular) epitope and is taken up during synaptic vesicle recycling. The 45 min incubation is sufficient to saturate all of the recycling vesicles (2), and thereby provides an estimate for the total recycling pool. The nonrecycling (reserve) pool of vesicles, which is larger than the recycling pool (Rizzoli and Betz, 2005), is not depicted here. To then obtain an estimate for the overall activity of the neurons at the particular time points, we applied Atto532-conjugated secondary nanobodies (NB) that target the Syt1 antibody, for 15 min (3). The nanobodies label only a subset of the vesicles, in proportion to the activity levels (4). The neurons were subsequently fixed, and were immunostained for synaptophysin (Syph) to label presynaptic compartments. To determine whether the assay indeed functioned, we blocked network activity with TTX, which only allows the Syt1 antibodies to bind to surface epitopes, or to spontaneously recycling vesicles (Truckenbrodt et al., 2018). ***B***, Exemplary images of neurons tested at 08:00 or 20:00, along with a TTX treatment example. Scale bar, 10 μm. ***C***, ***F***, The Syt1 and NB intensities were measured, with and without TTX treatment. Each symbol represents the average intensity of synapses in one image. *N* = 4 independent experiments; *n* = 20 images. Bar graph represents the mean ± SEM. The mean intensities were significantly lower on TTX treatment, in both ***C*** and ***E*** (two-way ANOVA test, followed by Tukey's multiple comparisons test, *p* < 0.0001 for both comparisons). ***D***, The Syt1 intensity over time. Symbols represent the mean ± SEM of each time point. *N* = 4 independent experiments; *n* = 20 images. No significant changes were observed (one-way ANOVA test, followed by Dunnett's multiple comparisons test, not significant for any comparisons). ***G***, The NB intensity over time. Symbols represent the mean ± SEM of each time point. The activity at 20:00 is significantly different compared with 08:00 (one-way ANOVA test, followed by Dunnett's multiple comparisons test, *p* = 0.0425 for 08 vs 20). *N* = 4 independent experiments; *n* = 20 images. ***E***, ***H***, We blocked network activity using TTX and then performed the same vesicle-labeling assay. The analysis of Syt1 and NB staining was performed as described in ***D*** and ***G***. Symbols represent mean ± SEM. *N* = 4 independent experiments; *n* = 20 images. No significant differences were found when using one-way ANOVA tests, followed by Dunnett's multiple comparisons test. ***I***, In order to test whether the variations in presynaptic activity are accompanied by morphologic or size changes, neurons were immunostained at different time points for the presynaptic marker Syph and for the postsynaptic marker Homer1. Scale bar, 5 μm. ***J***, To determine whether the number of synapses vary throughout 24 h, we measured the numbers of Syph- and Homer1-positive objects from the images shown in Figure 2. Graphs represent the number of objects (synapses) per image (mean ± SEM). *N* = 4 independent experiments; *n* = 20 images. No significant differences were found when using one-way ANOVA tests, followed by Dunnett's multiple comparisons test. ***K***, The intensity of Syph and Homer1 stainings over time (mean ± SEM; *N* = 4 independent experiments; *n* = 20 images). The intensities at 02:00 for both stainings are significantly higher compared with the stainings at 08:00 (one-way ANOVA test, followed by Dunnett's multiple comparisons test). The first time point was the reference for the multiple comparison. *p* = 0.0153 for Syph. *p* = 0.0023 for Homer1. *p < 0.05. **p < 0.005. ****p < 0.0001. ns, not significant.

**Figure 4.**
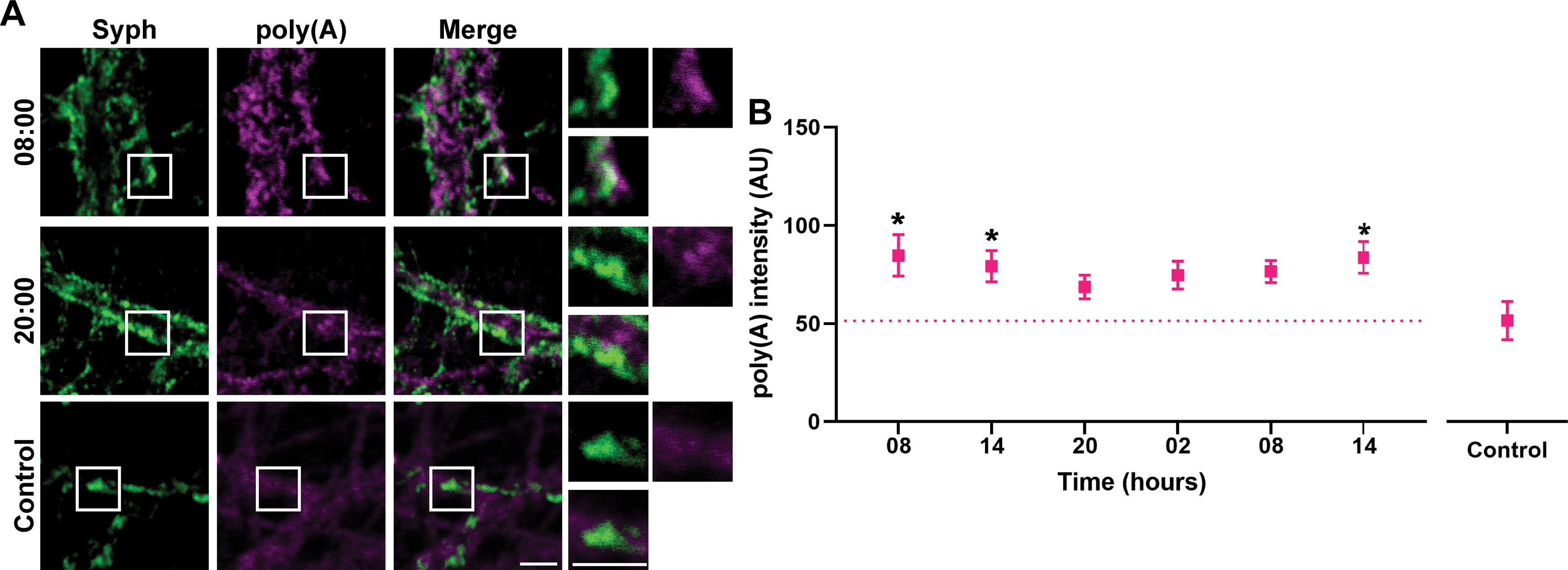
The amount of mRNA at the synapse is dynamic throughout 24 h. ***A***, To monitor the changes in the mRNA amounts throughout the day and night, we labeled the poly(A) tail of mRNAs with Atto647N-conjugated oligo(dT), at DIV18. We also immunostained the neurons for Syph, to determine synaptic locations. As a background staining control, we relied on Atto647N-conjugated oligo(dA). Exemplary images for the stainings at 08:00 and 20:00 are shown, along with negative controls. Scale bars, 2.5 μm. ***B***, The synaptic signals were determined in ROIs centered on the Syph spots, and broadened by 200 nm in each direction, to also include potential postsynaptic sites. Symbols represent mean ± SEM of an image. *N* = 3 independent experiments; *n* = 18 images. The statistical significance was calculated with a Kruskal–Wallis test, followed by Dunn's multiple comparisons test. The negative control signal was the reference for the multiple comparison. *p* = 0.225, Control versus 08. *p* = 0.0493, Control versus 14. *p* = 0.0186, Control versus 14. *p < 0.05.

**Figure 5.**
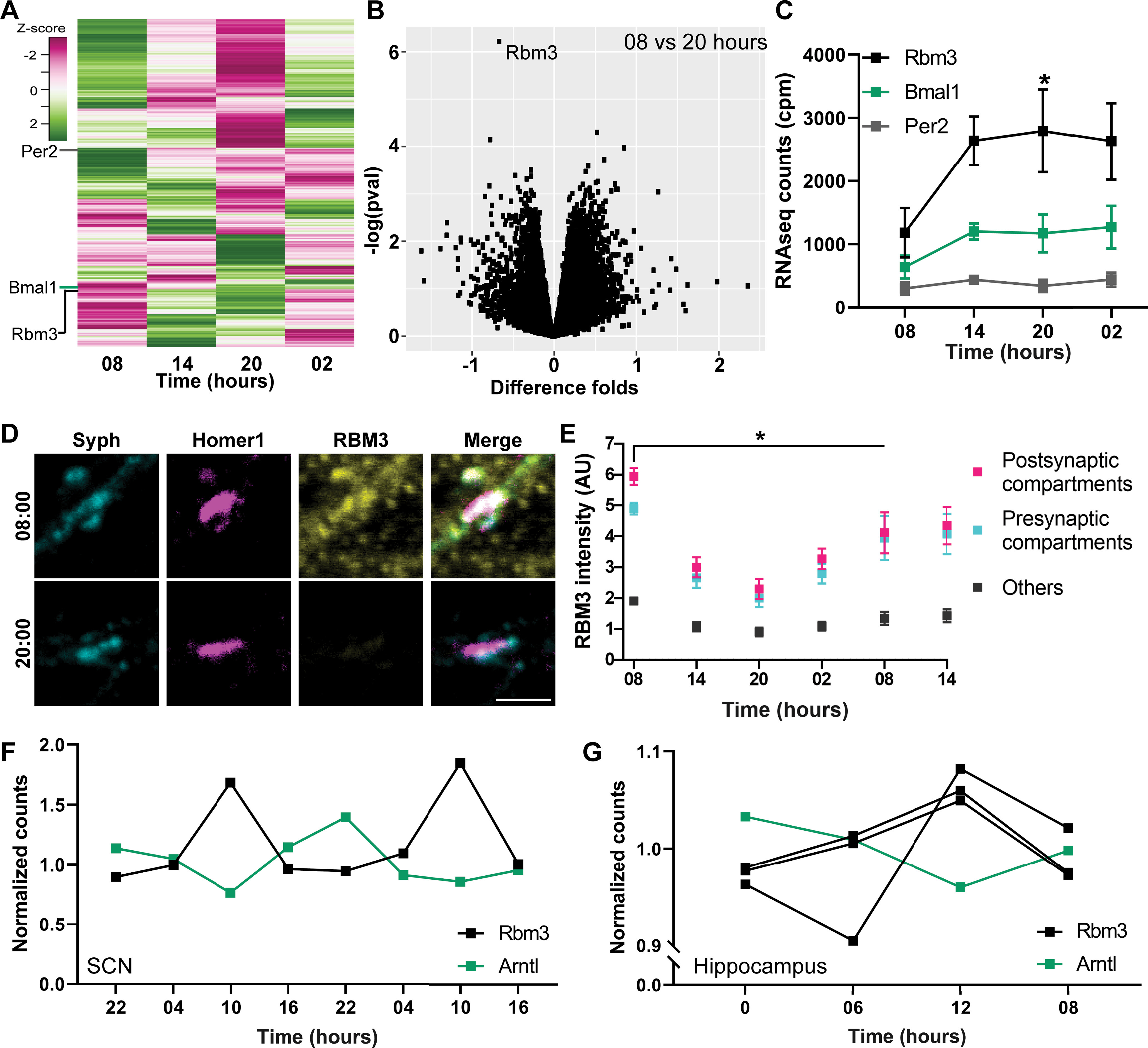
RBM3 abundance varies throughout 24 h in the hippocampal cultures. ***A***, To identify candidate genes that are responsible for the activity changes observed in the previous sections, we sequenced mRNAs collected from the cultures at different time points. Each row represents a gene. For expression profiling, a *z* score was calculated, and was scaled between −3 and 3. The *z* score describes the distance of the expression value at a given time point to the mean of the overall gene expression (for the gene set enrichment analysis, see Table 1). ***B***, A volcano plot was generated to visualize the significance of the changes together with the fold difference. The graph plots –log(*p* value) versus the ratio between the amount of mRNAs that were collected at 08:00 and 20:00. The *p* values are not adjusted for multiple comparisons in this graph. After correction for multiple comparisons (using the R package Limma) (Ritchie et al., 2015), only the changes in RBM3 appear significant. ***C***, The expression pattern of Rbm3 is plotted along with that of the core clock genes Per2 and Bmal1. Symbols represent the mean ± SEM of the counts per million (cpm) mapped reads. *N* = 6 independent experiments. *p* = 0.0073. ***D***, To measure the RBM3 protein abundance, we immunostained it at different time points, along with Syph and Homer1, to identify synapses. Scale bar, 2.5 μm. ***E***, The intensities of RBM3 at the presynaptic and postsynaptic locations, as well as in all other cell regions, were calculated (mean ± SEM). The statistical significance was calculated with a Kruskal–Wallis test, followed by Dunn's multiple comparisons test. The first time point was the reference for the multiple comparison. *N* = 4 independent experiments; *n* = 20 images. *p* values for presynaptic compartments (08 vs 08, 14, 20, 02, 08): 0.0008, <0.0001, 0.0039, 0.0274, 0.1660; for postsynaptic compartments (08 vs 08, 14, 20, 02, 08): 0.0001, <0.0001, 0.0011, 0.0085, 0.0617; for others (08 vs 08, 14, 20, 02, 08): 0.0014, <0.0001,0.0031, 0.0285, 0.1230. *p < 0.05. ***F***, Graph represents the median normalized expression pattern of Arntl and Rbm3 throughout the day and night in the SCN (GSE70391; *p* = 0.0000 for Rbm3, *p* = 0.0026). ***G***, Graph represents the median normalized expression pattern of Arntl and 3 transcripts of Rbm3 throughout the day and night in the hippocampus tissue (GSE66875) (Renaud et al., 2015): *p* = 0.0017 for Rbm3, *p* = 0.0897 for Rbm3, *p* = 0.0040 for Rbm3, and *p* = 0.0017 for Arntl. The circadian rhythmicity was confirmed via the MetaCycle (Wu et al., 2016). All time series shown have a significant circadian rhythm.

**Figure 6.**
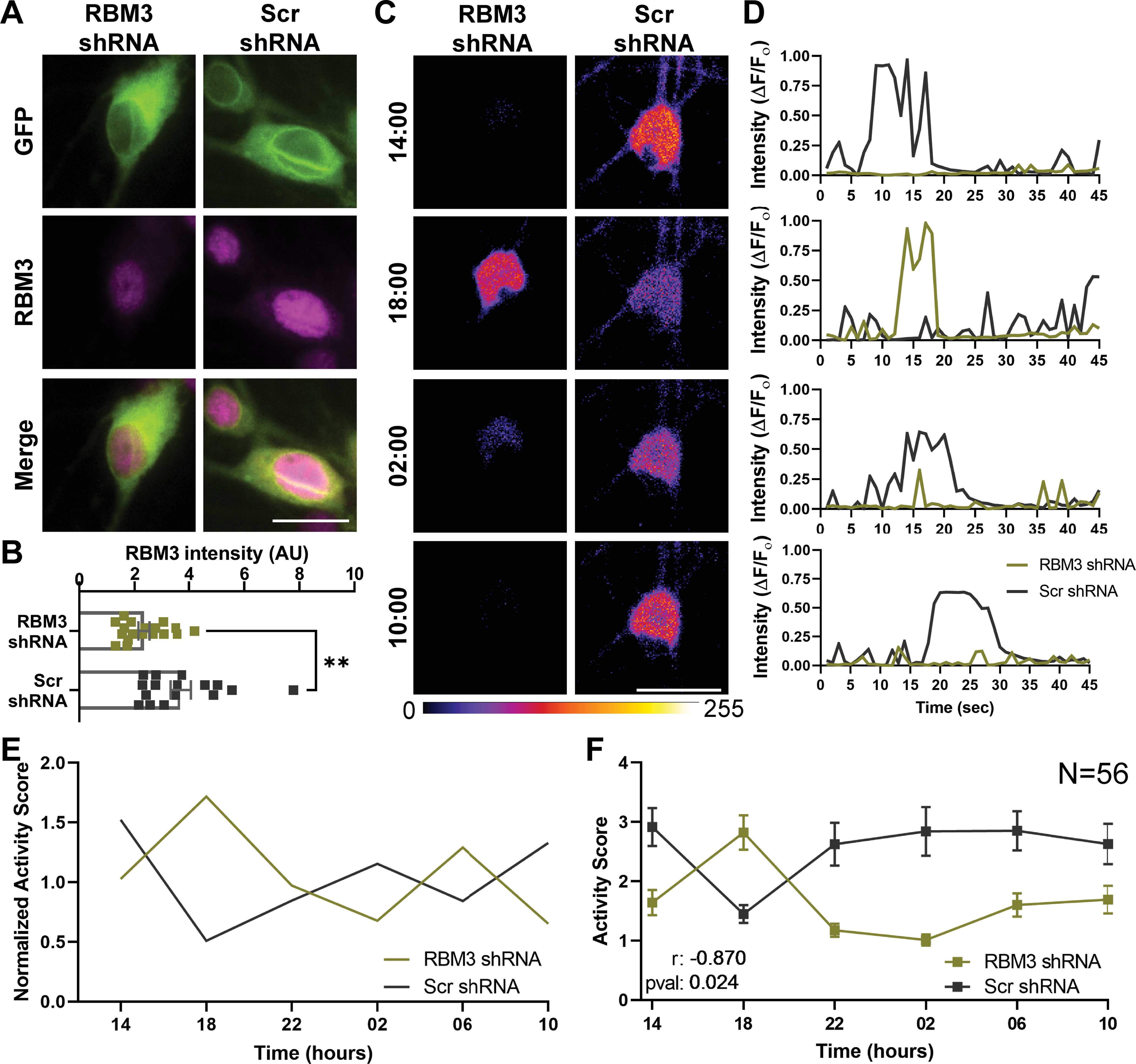
Knocking-down RBM3 alters the neuronal activity pattern throughout 24 h. ***A***, We knocked down RBM3 in the cultures by shRNA expression (see Materials and Methods). Alternatively, a scrambled shRNA was expressed (Scr), as a control. Exemplary RBM3 stainings are shown. The reporter for the shRNA virus is GFP. Scale bar, 10 µm. ***B***, To reveal how RBM3 shRNA is affecting the RBM3 abundance, we performed immunostaining on shRNA treatments. Each dot represents the mean of an image. Error bar indicates mean ± SEM. *N* = 3 independent experiments; *n* = 15 images. A significant difference was detected (Mann–Whitney test): *p* = 0.0021. **p < 0.005. ***C***, To determine the firing patterns, we performed Ca^2+^ experiments on RBM3 knocked down neurons, exactly as in Figure 1. Scale bar, 50 μm. ***D***, The signals over the entire 45 s recordings from the cells shown in ***C*** are plotted, at the four different time points. ***E***, The activity score is plotted for the two exemplary neurons, normalized to their respective medians. ***F***, To reveal the average firing patterns, 56 neurons were measured in each condition, from four independent experiments (with 2-4 different wells measured per experiment; mean ± SEM). To calculate the correlation between two firing patterns, we performed a Pearson's correlation test. A significant anticorrelation was observed (*r* = −0.870, *p* = 0.024).

**Figure 7.**
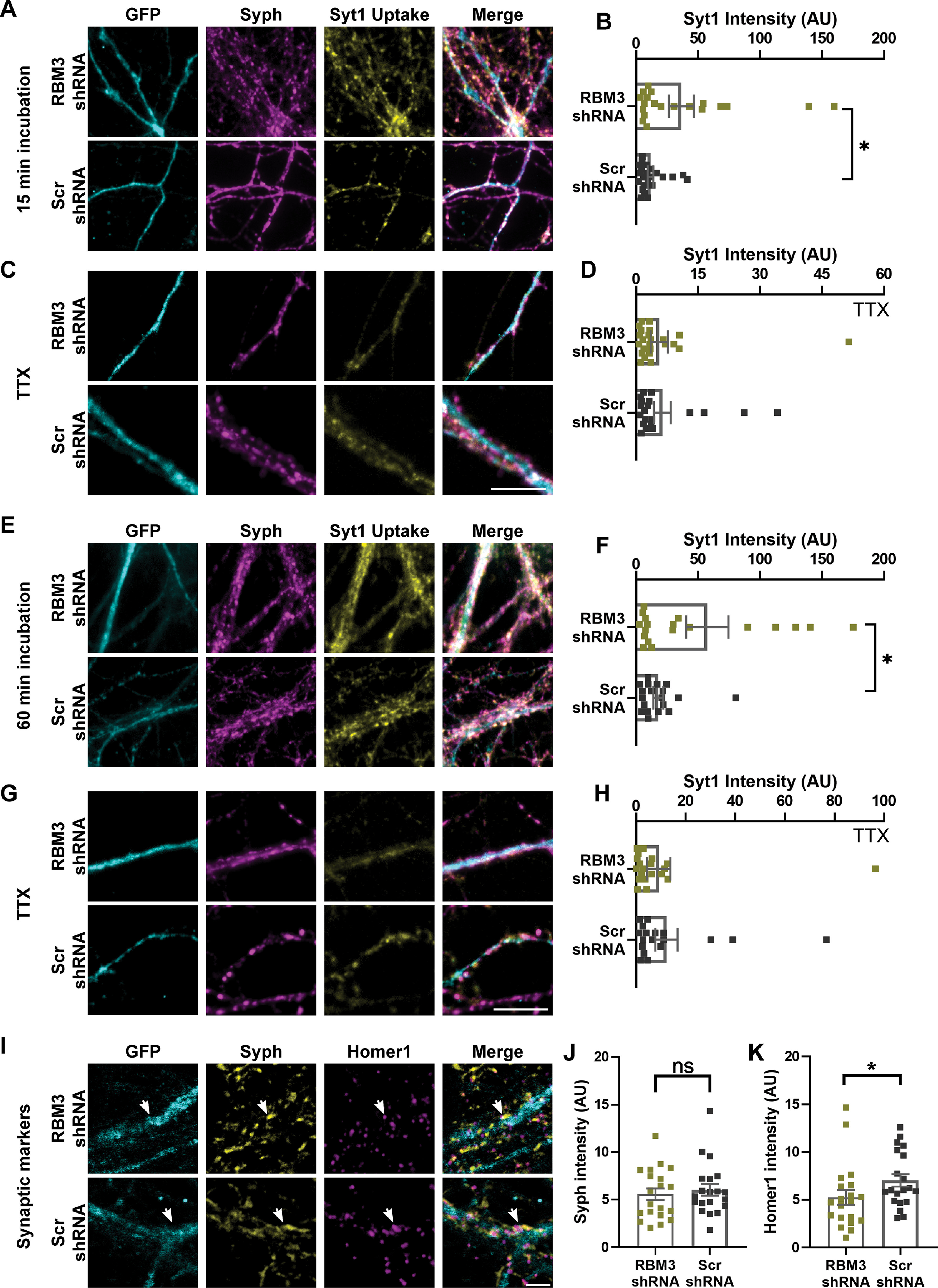
Synaptic activity and morphology are modified on RBM3 shRNA treatment. ***A***, ***E***, To measure synaptic vesicle recycling, we relied on the Syt1 uptake assay, applying the antibodies for 15 or 60 min, in different experiments. The assays were performed on DIV18 neurons, at 18:00. GFP is used as a reporter for the expression of shRNA or a scrambled control sequence. Typical images are shown. Scale bar, 20 µm. ***B***, ***F***, The Syt1 intensity in 15 and 60 min incubations were measured. Symbols represent the mean of each image. Error bar indicates mean ± SEM. *N* = 4 independent experiments; *n* = 20 images. The recycling vesicle pool size (measured with 60 min incubations) as well the synaptic activity (measured with 15 min incubations) are significantly larger for the RBM3 knockdowns (Mann–Whitney tests). ***B***, *p* = 0.0138. ***F***, *p* = 0.0350. ***C***, ***G***, We blocked network activity using TTX and then performed the same vesicle-labeling assay as in ***A*** and ***E***. Typical images are shown. Scale bar, 20 µm. ***D***, ***H***, The Syt1 intensity was measured in the TTX condition in 15 and 60 min incubations. Symbols represent the mean of each image. Error bar indicates mean ± SEM. *N* = 4 independent experiments; *n* = 20 images. A quantification of the signals revealed no significant differences (Mann–Whitney tests). ***D***, *p* = 0.8065. ***H***, *p* = 0.6362. ***I***, To monitor changes in the size or morphology of synapses, neurons were immunostained for Syph (presynaptic marker) and Homer1 (postsynaptic marker). Scale bar, 2.5 μm. White arrows indicate the synapses. ***J***, ***K***, The intensity of the Syph and Homer1 stainings, respectively. Symbols represent the mean intensity of each image. Error bar indicates mean ± SEM. *N* = 4 independent experiments; *n* = 20 images. The Homer1 intensity of RBM3 KD is significantly lower than in the controls (Mann–Whitney tests). ***J***, *p* = 0.6980. ***K***, *p* = 0.0402. *p < 0.05. ns, not significant.

**Figure 8.**
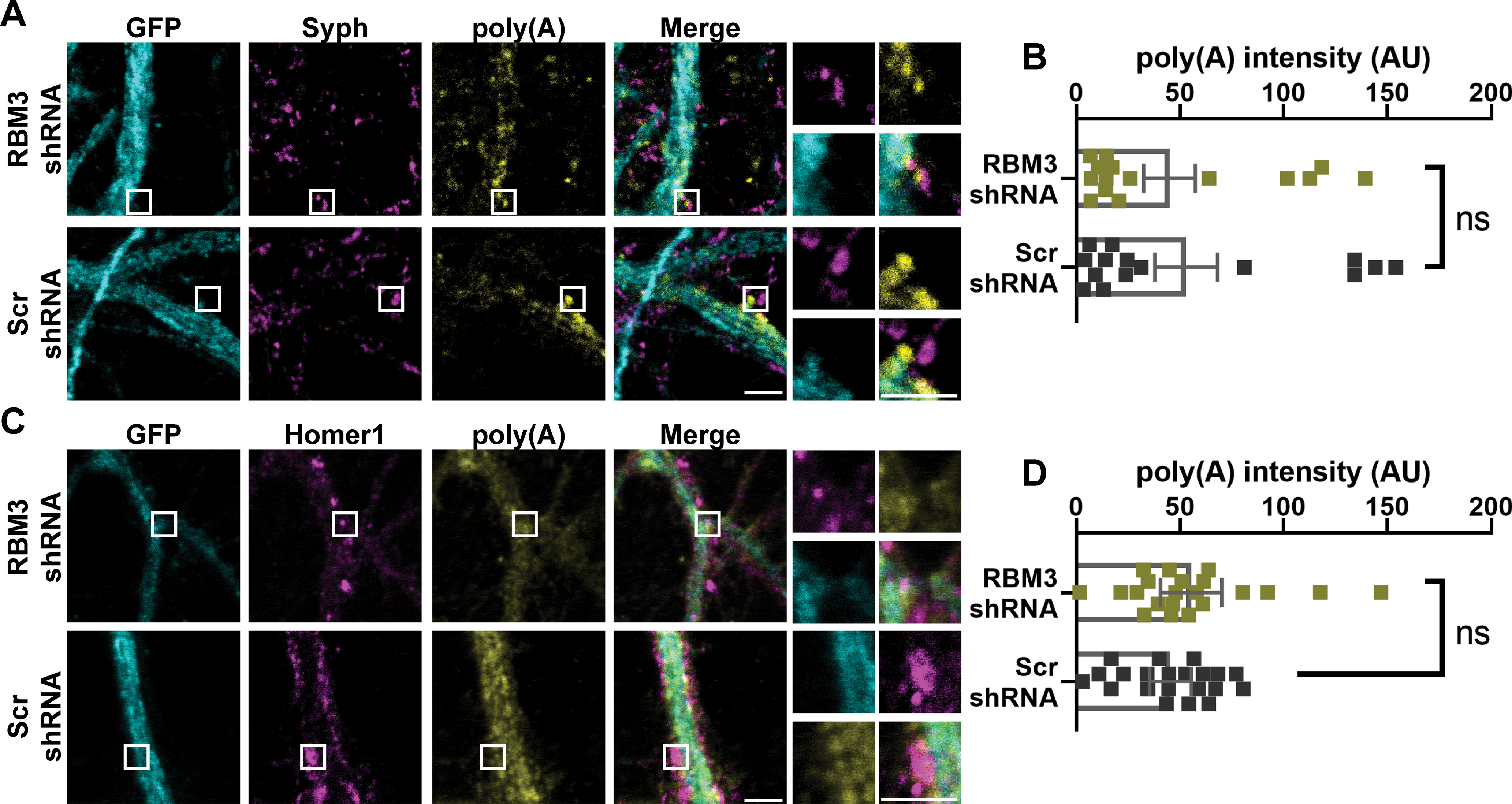
The synaptic mRNA levels are not affected by RBM3 knockdowns. ***A***, ***C***, To determine the effects of RBM3 on mRNA levels at the synapse, FISH with oligo(dT) was performed with shRNAs as in Figure 3 together with immunostaining for the presynaptic and postsynaptic markers, Syph and Homer1, respectively. GFP is a reporter for shRNA virus. Scale bar, 2.5 µm. ***B***, ***D***, An analysis of the FISH signal at synaptic compartments indicates no significant difference between RBM3 knockdowns and controls (Kruskal–Wallis, followed by Dunn's multiple comparisons test). Each dot represents the mean of an image. Error bar indicates mean ± SEM. *N* = 3 independent experiments and *n* = 15 images for Syph staining; *p* = 0.9349. *N* = 4 independent experiments and *n* = 20 images for Homer1 staining; *p* = 0.5499. ns, not significant.

**Figure 9.**
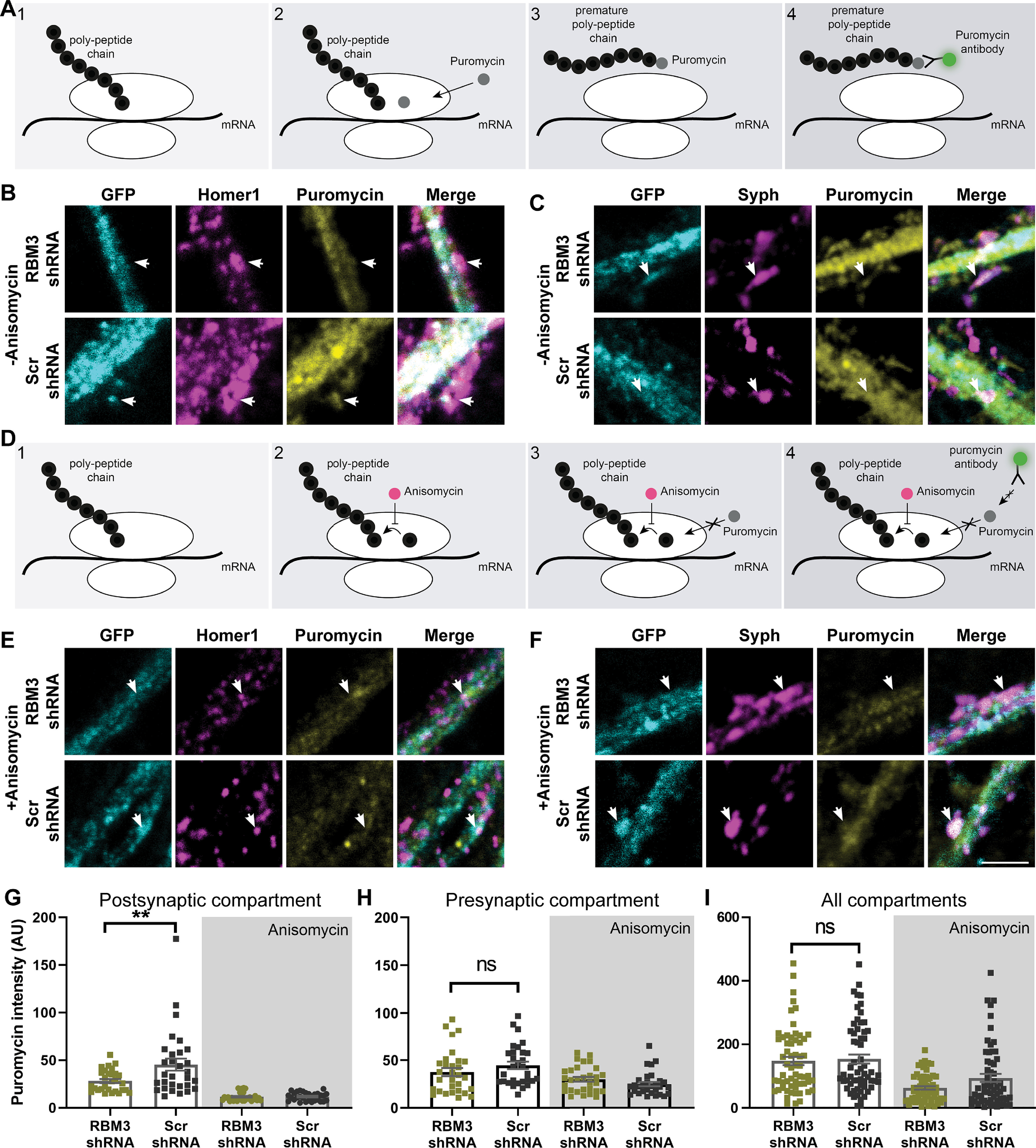
RBM3 knockdown decreases translation at the postsynapse. ***A***, To measure local translation at the synapse (1), we used the puromycin assay. Puromycin binds to the P site in the ribosome (2). It incorporates itself into the polypeptide chain and releases the polypeptide chain prematurely (3). A subsequent immunostaining for puromycin (4) enables an estimation of the amount of local translation. As a negative control, we treated the cultures with anisomycin (see ***D***), which prevents puromycin binding. ***B***, ***C***, Puromycin immunostainings are shown, along with Homer1 or Syph stainings, to indicate postsynaptic and presynaptic sites. Scale bars, 2.5 µm. ***D***, To measure the puromycin effect after protein synthesis inhibition, (1) we used anisomycin as a negative control before puromycin treatment. Anisomycin blocks the amino acid transfer to the polypeptide chain (2). Therefore, puromycin cannot be incorporated into the polypeptide chain (3). A subsequent immunostaining for puromycin (4) enables an estimation of the amount of puromycin incorporation, which have overcome the anisomycin effect. ***E***, ***F***, As a negative control, we performed the puromycin assay together with anisomycin as in ***D***. Typical images for the negative control of the puromycin assay are shown together with Homer1 (postsynaptic marker) or Syph (presynaptic marker) staining. Scale bar, 2.5 µm. ***G***, Puromycin staining intensities for the Homer1 areas are shown. Each dot represents the mean of an image. Error bar indicates mean ± SEM. *N* = 4 independent experiments; *n* = 20 images. The RBM3 knockdowns show significantly less translation at postsynapses (one-way ANOVA, followed by Dunnett's multiple comparisons test). *p* = 0.0029. **p < 0.005. ***H***, Puromycin staining intensities for the Syph areas are shown. Each dot represents the mean of an image. Error bar indicates mean ± SEM. *N* = 4 independent experiments; *n* = 20 images. The RBM3 knockdowns appear to lower translation, but the overall levels in presynapses are too close to the negative controls (anisomycin) for this difference to be determined with precision (the difference was not significant when tested by a Kruskal–Wallis test, followed by Dunn's multiple comparisons test). *p* = 0.0536. ***I***, An analysis of the global puromycin levels in RBM3 knockdowns and controls. Each dot represents the mean of an image. Error bar indicates mean ± SEM. *N* = 4 independent experiments. No significant difference was observed by a Kruskal–Wallis test, followed by Dunn's multiple comparisons test. *p* = 0.9719. ns, not significant.

**Figure 10.**
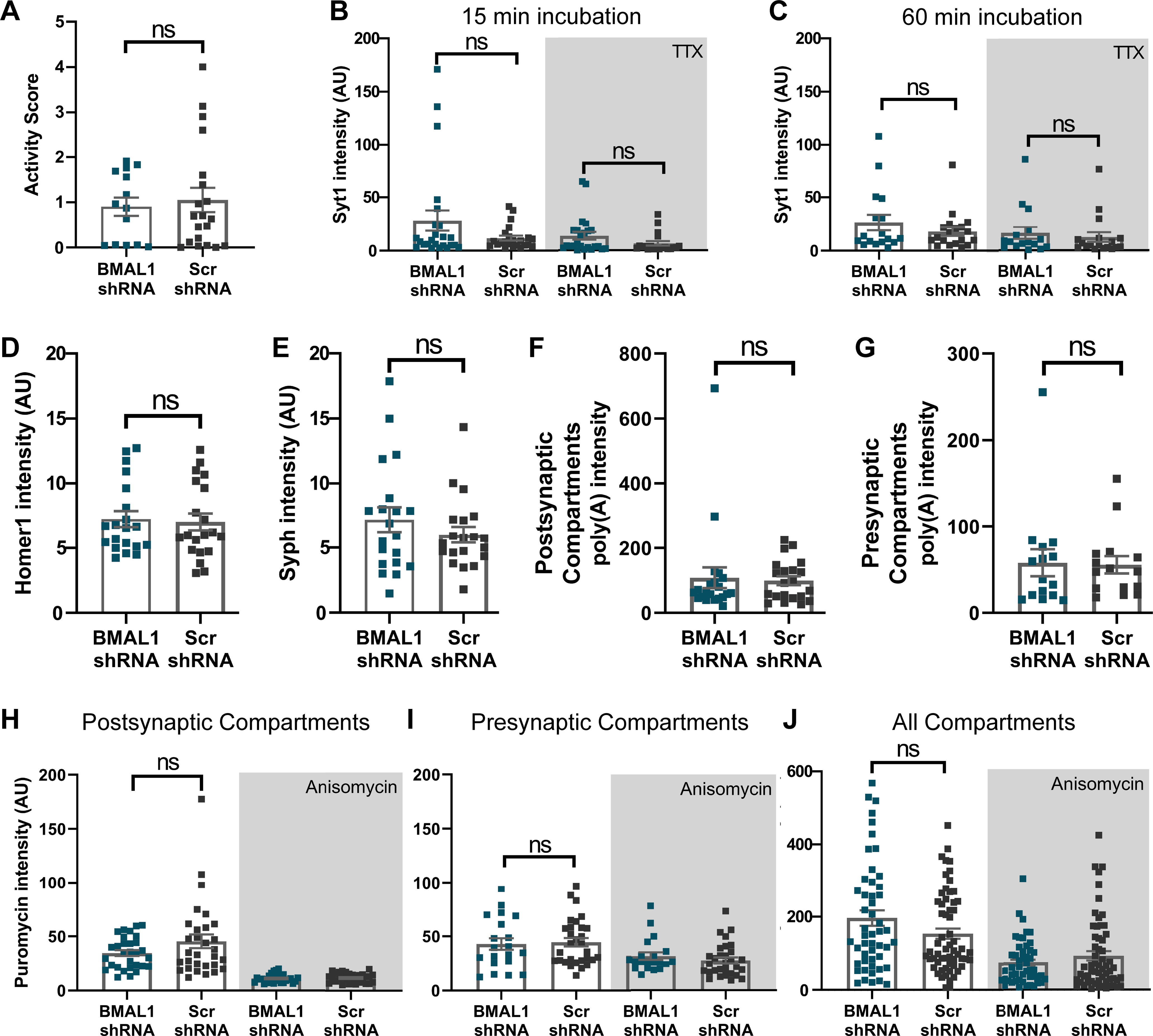
The effect of BMAL1 on neurons. ***A***, To measure the contribution of the molecular clock in the primary hippocampal culture, we performed same assays with BMAL1 KD neurons. Again, we knocked down BMAL1 in the cultures by shRNA expression (see Materials and Methods). Alternatively, a scrambled shRNA was expressed (Scr), as a control. To determine the firing patterns, we performed Ca^2+^ experiments, exactly as in Figure 6. The signals over the entire 5 min recordings from the cells shown are plotted. We selected maximum 5 neurons from one coverslip, and performed 4 coverslips per experiment. *N* = 3 independent experiments (Mann–Whitney test, *p* = 0.7621). ***B***, ***C***, We performed Syt1 assay as described in Figure 7. The Syt1 intensity was measured. Symbols represent the mean of each image. Error bar indicates mean ± SEM. *N* = 4 independent experiments; *n* = 20 images. The recycling vesicle pool size (measured with 60 min incubations) as well the synaptic activity (measured with 15 min incubations) do not show difference for the BMAL1 knockdowns (Mann–Whitney tests, *p* = 0.5906 for 15 min incubation, *p* = 0.1177 for TTX with 15 min incubation, *p* = 0.73 066 for 60 min incubation, *p* = 0.4400 for TTX with 60 min incubation). ***D***, ***E***, To monitor changes in the size or morphology of synapses, neurons were immunostained for Syph and Homer1. Graphs show the intensity of the Syph and Homer1 stainings, respectively. Symbols represent the mean intensity of each image. Error bar indicates mean ± SEM. *N* = 4 independent experiments; *n* = 20 images. The Homer1 intensity of BMAL1 KD is not different from in the controls (Mann–Whitney tests, *p* = 0.821 for Homer1 and *p* = 0.5291 for Syph staining). ***F***, ***G***, To determine the effects of BMAL1 on mRNA levels at the postsynapse (Homer1 as a marker) and presynapse (Syph as a marker), respectively. An analysis of the FISH signal indicates no significant difference between BMAL1 knockdowns and controls (Mann–Whitney test, *p* = 0.4697 for postsynaptic and *p* = 0.6827 for presynaptic compartments). Each dot represents the mean of an image. Error bar indicates mean ± SEM. *N* = 3 independent experiments; *n* = 20 images. ***H-J***, To measure local translation at the postsynapse, we used the puromycin assay as described in Figure 9. Puromycin staining intensities are shown, calculated for the Homer1, Syph, and all areas, respectively. Each dot represents the mean of an image. Error bar indicates mean ± SEM. *N* = 4 independent experiments; *n* = 20 images. The BMAL1 knockdowns do not show significant change in local translation (one-way ANOVA, followed by Dunnett's multiple comparisons test, *p* = 0.1233 for postsynaptic, *p* = 0.4440 for presynaptic compartments; Kruskal–Wallis test, followed by Dunn's multiple comparisons test, *p* = 0.8721 for all compartments). ns, not significant.

To find correlations between two groups, Pearson's correlation was used (see Figs. 1*E*, 2*B*, 6*F*). For long-term calcium imaging where individual neurons were traced, the Friedman test followed by Dunn's multiple comparisons test was used (see Fig. 1*D–G*). To determine the significant changes in time-series nonparametric data, the Kruskal–Wallis test followed by Dunn's multiple comparisons test was used (see Figs. 4*B*, 5*E*, 8*B*,*D*, 9*H*,*I*, 10*J*). For parametric time-series data, the one-way ANOVA test followed by Dunnett's multiple comparisons test was used (see Figs. 3*D*,*G*,*E*,*H*,*J*,*K*, 9*G*, 10*H*,*I*). For analysis between groups, we used Mann–Whitney test (see Figs. 6*B*, 7*B*,*D*,*F*,*H*,*J*,*K*, 10*A-G*). For multiple comparisons, we used two-way ANOVA test followed by Tukey's multiple comparisons test (see Fig. 3*C*,*F*). Tests were performed by GraphPad Prism software version 8.3.0 (GraphPad Software).

## Results

### Neuronal activity changes throughout 24 h in dissociated hippocampal cultures

Primary hippocampal cultures are widely used, as they are relatively simple to prepare and maintain most of the important functional features of the *in vivo* neurons (Dotti et al., 1988). We used them here, relying on a classical protocol (Banker and Cowan, 1977) that dissociates the hippocampi of newborn rats and results in mixed glia and neuron cultures. Most of the neurons are glutamatergic (>90%) (Benson et al., 1994) and have a mature morphology and synapse development already at ∼12 DIV.

To determine whether primary hippocampal cultures show differences in their electrical activity in dependence on the time of day/night, we performed long-term calcium imaging, using a genetically encoded calcium indicator, NeuroBurst (Sartorius). To sample culture activity regularly, we imaged the neurons (starting at DIV18) every 4 h, for 45 s (Fig. 1*A*). This enabled us to obtain a fluorescence-based measure of the activity of the individual neurons at the particular time points (Fig. 1*B*), which we termed “activity score.” Individual neurons exhibited changes in the activity score throughout the day and night, with the examples shown in Figure 1*B* having strong peaks at 6:00 and 14:00. Such changes in activity were observed for all neurons investigated (see a selection in Fig. 1*C*).

To test whether the activity of the different neurons was synchronized, we performed this experiment with four different culture preparations, in which we tracked 156 different neurons. Their average activity score showed significant changes throughout the measurement (Fig. 1*D*), with activity being high at 14:00, dropping until ∼22:00, and rising again after 02:00, before dropping again for several hours, and finally rising one more time before the end of our measurements. Randomizing the timing of the individual neuronal measurements eliminates all significant changes (Fig. 1*G*,*H*), which suggests that the results obtained here are unlikely to be because of chance, but are rather because of synchronous culture activity. The pattern observed does not conform to a precise 24 h pattern (Fig. 1*D*). Two possible interpretations could be made. First, the cultures exhibit their own pattern of activity, which does not relate to a 24 h rhythm. Second, the activity of the individual neurons does conform to a 24 h rhythm, but they are partially desynchronized, so that a 24 h rhythm is no longer observed at the whole culture level, especially when averaging results across different cultures, as in Figure 1*D*. The second interpretation appeared probable, since 24 h patterns are difficult to maintain with precision even in SCN cultures (Honma et al., 1998). Interestingly, the first 32 h of the recordings overlapped well with the next 32 h (Fig. 1*E*). Similar observations were made for younger cultures (DIV10; Fig. 1*F*). Again, the pattern did not seem to conform to a simple 24 h cycle.

To test this in more detail, we analyzed whether the activity patterns of individual neurons correlated significantly to the 24 h pattern of a bona fide molecular clock gene. We expressed in our cultures a destabilized GFP molecule, relying on the promoter of the clock gene BMAL1. The fluctuations in the GFP amounts, which report the BMAL1 promoter activity, conformed to a 24 h cycle, with peaks at night and lower values during the day (Fig. 2). In parallel, we analyzed the activity patterns of the GFP-expressing neurons (Fig. 2). We found that their activity patterns correlated significantly (albeit negatively) to the BMAL1 promoter activity. This implies that the activity of individual neurons in these cultures can be seen as exhibiting a 24 h rhythm, albeit this is difficult to observe when averaging many neurons and independent cultures, because of a partial desynchronization.

### The dynamics of the synaptic vesicles also change throughout 24 h

Oscillations in neuronal activity should also be reflected at the synaptic level, especially in the synaptic vesicle dynamics. The vesicle behavior can be analyzed with precision by using antibodies that detect the luminal (intravesicular) domain of the vesicular calcium sensor synaptotagmin 1 (Syt1) (Matteoli et al., 1992; Kraszewski et al., 1995). The antibodies are taken up by synaptic vesicles during their recycling, since they expose the luminal epitopes during exocytosis, and thus enable the antibody to penetrate into the vesicles, and to be endocytosed (Fig. 3*A*). We incubated the cultures every 6 h with fluorescently conjugated Syt1 antibodies for 45 min. This time interval is sufficient to label (saturate) all active synaptic vesicles, and therefore to provide a measure of the active vesicle pool size (Truckenbrodt et al., 2018). We then applied to the cultures fluorescently conjugated nanobodies that recognize the Syt1 antibodies, for 15 min. The nanobodies bind Syt1 antibodies that are exposed to the surface during vesicle activity (Fig. 3*A*). This short incubation interval does not saturate all binding sites (Truckenbrodt et al., 2018), and therefore provides a measure of synaptic activity at the respective time point, rather than a measure of the vesicle pool size. To confirm the validity of this assay, we compared normal neurons with neurons in which network activity was blocked using the Na^+^ channel inhibitor TTX. TTX blocked active vesicle recycling, and therefore reduced both the antibody and nanobody stainings (Fig. 3*B*,*C*,*F*), as expected.

These measurements suggested that the size of the actively recycling vesicle pool is relatively constant throughout 24 h (Fig. 3*D*), but the synaptic activity, as measured by the nanobody intensity, exhibits significant differences (Fig. 3*G*). None of these measurements showed any changes in cultures maintained constantly in TTX, as expected (Fig. 3*E*,*H*).

Overall, these experiments confirm the idea that, at the synapse level, neurons show changes in their activity patterns throughout 24 h. As these measurements only targeted the active vesicles, which make up only ∼50% of all vesicles (Rizzoli and Betz, 2005), we sought to also obtain a measurement of the entire vesicle pool, by immunostaining the synapses at different time points, relying on the vesicle marker synaptophysin (Takamori et al., 2006). This again showed changes during 24 h, with a substantial increase at 02:00 (Fig. 3*I*,*K*). We observed similar behavior for a marker of the postsynaptic density, Homer1 (Fig. 3*K*). No changes could be detected in the number of synapses (Fig. 3*J*). These results suggest that not only neuronal and synapse activity, but also synapse size, depends on the time of day/night.

### Synaptic mRNA amounts are subject to change over 24 h

Along with brain activity, brain metabolism also changes throughout the day and night cycle, including aspects as transcription and translation, which have been shown to exhibit strong circadian rhythmicity (Noya et al., 2019). We therefore proceeded to test whether such changes could also be observed in cultured hippocampal neurons. We analyzed the mRNA levels in the cultures, relying on FISH, performed with fluorescently conjugated oligonucleotides containing multiple thymidine (dT) moieties. These label specifically the polyadenylated tails of mRNAs, and showed measurable signals throughout the cells, including synaptic areas (Fig. 4*A*). We analyzed the FISH signals and found that they changed throughout the 24 h (Fig. 4*B*). Nevertheless, these results demonstrate that dynamic changes of the mRNA levels take place over time in disassociated hippocampal cultures.

### The abundance of RBM3 changes over 24 h, especially at synapses

To determine the molecular mechanisms responsible for the changes in neuronal activity, synapse morphology, and mRNA amounts throughout 24 h, we analyzed the transcriptome of the cultures at different times of day/night, using mRNA sequencing (mRNAseq) (Fig. 5*A*, *N* = 6 independent experiments). Although several overall changes could be seen among the different sets of genes, relating to processes, such as synaptic transmission and neuronal morphogenesis (Table 1), only one transcript showed a significant differential expression when the results from six different culture preparations were combined (Fig. 5*B*): RNA-binding motif 3 (Rbm3). This molecule showed the same general pattern of expression as bona fide clock genes, such as like Bmal1 and Per2 (Fig. 5*C*), but its variation among different cultures was small enough to result in significant differences between the time points, unlike Bmal1 or Per2. We assume that the desynchronization between different cell cultures is strong enough to mask the rhythmicity of Bmal1 or Per2, albeit at least the former is clearly exhibiting changes over 24 h in the cultured neurons (Fig. 2).

Before pursuing this idea, we confirmed that its abundance is indeed cyclic in neurons. RBM3 immunostainings revealed that the protein has profound oscillations throughout 24 h, with minima in the evening and maxima around 08:00 (Fig. 5*D*,*E*). These changes were far more profound in synapses than in other compartments (Fig. 5*E*), which again suggests that this molecule may be involved in synaptic function.

RBM3 is a cold-shock protein that is involved in translation, and has been published as a potential biomarker for breast and colon cancer because of this role (Sureban et al., 2008). However, RBM3 has also been connected to brain function, since it was discovered that it protects synapses from hypothermia (Peretti et al., 2015). More interestingly, it controls the alternative polyadenylation of core clock genes, which explains its connection to the circadian clock (Liu et al., 2013). A systematic transcriptomics analysis of the molecular circadian rhythm indicates that Rbm3 is a rhythmically expressed transcript in the SCN tissue (Yan et al., 2008). To confirm this, we also surveyed circadian transcriptomics datasets that are available online at the Gene Expression Omnibus repository (Fig. 5*F*,*G*). We found that RBM3 has a rhythmic expression in the SCN (GSE70391). Moreover, Rbm3 exhibits rhythmic expression throughout the day and night cycle in the hippocampus tissue *in vivo* (GSE66875) (Renaud et al., 2015). RBM3 appeared therefore as a strong candidate molecule, which may be involved in organizing daily (24 h) patterns of neuronal activity.

### RBM3 controls the firing pattern in primary hippocampal cultures

To determine whether RBM3 affects neuronal activity, we resorted to long-term calcium imaging (as in Fig. 1), in cultures subjected to RBM3 knockdown, or to the transfection of a scrambled oligonucleotide control. The RBM3 knockdown reduced the amounts of the protein significantly (Fig. 6*A*,*B*), albeit not completely. When analyzing in parallel the calcium signals from the knockdown cultures and their controls, it became obvious that activity could still be detected in both conditions (Fig. 6*C*,*D*), but that the patterns were different. The RBM3 knockdowns had peaks of activity at precisely the time points when the control cultures had their minimal activity (Fig. 6*E*,*F*). This relation was significant (Fig. 6*F*), implying that RBM3 has an important role in regulating the pattern of neuronal activity.

To test whether these effects also translated to synaptic vesicle activity, we relied on the Syt1 uptake assay used in Figure 3. We chose the time point that exhibited the highest difference in activity in calcium imaging (18:00; Fig. 6), and incubated the cultures with Syt1 antibodies for 15 min, to determine the activity levels (Fig. 7*A–D*), or for 60 min, in different coverslips, to measure the total size of the actively recycling vesicle pool (Fig. 7*E–H*). As cultures undergoing the knockdown treatment may be more fragile than unmodified cultures, we avoided the assay relying on mixtures of nanobodies and antibodies (from Fig. 3*A*), which involves multiple buffer changes that may harm the cultures, and we simply relied on separate 15 or 60 min incubations. Both measurements showed significant differences, with RBM3 knockdown enhancing synaptic function (Fig. 7*B*,*F*), in agreement with the change in activity observed at 18:00 in calcium imaging (Fig. 6). Performing these measurements in the presence of TTX, which blocks network activity, resulted in no significant differences between the knockdowns and the controls (Fig. 7*D*,*H*).

Finally, immunostainings for synaptophysin or Homer1, performed to determine the synapse size (as in Fig. 3*I–K*), suggested that RBM3 knockdown significantly influences the postsynapse size (Fig. 7*I–K*). Overall, these results demonstrate that RBM3 is strongly involved in the changes of synaptic and neuronal function throughout 24 h.

### RBM3 controls local translation at the postsynapse

As mRNA amounts varied throughout 24 h (Fig. 4), and as RBM3 is an mRNA binding protein, we next sought to determine whether the RBM3 knockdown influences the mRNA levels in synapses, where the highest RBM3 oscillations were observed (Fig. 5). We repeated the FISH experiments performed in Figure 4, either in RBM3 knockdown neurons or in controls (Fig. 8). No significant changes could be observed: neither when relying on synaptophysin as a synaptic marker (Fig. 8*A*,*B*), nor when relying on the postsynaptic marker Homer1 (Fig. 8*C*,*D*). This suggests that RBM3 does not influence the synaptic mRNA levels, at least not sufficiently for detection with this FISH assay.

As RBM3 has been strongly linked to translation (Dresios et al., 2005; Smart et al., 2007), we next analyzed its potential influence on this process. We relied on an assay that reports the translation sites, the so-called puromycin assay (Hafner et al., 2019). Puromycin is an antibiotic that binds to the P site of the ribosome and incorporates itself into the polypeptide chain. This results in the polypeptide chain being released from the ribosome prematurely (Fig. 9*A*), thereby stopping the translation process. A subsequent immunostaining with a specific puromycin antibody reports all of the stopped translation sites, thereby providing an accurate estimate of ongoing translation in the particular cellular area. We combined this assay with the RBM3 knockdown (Fig. 9*B*) and found that this treatment significantly reduced local translation in postsynapses (Fig. 9*G*). The translation levels remaining in RBM3 knockdowns were close to the background levels, measured by pretreating the cultures with anisomycin, an antibiotic that halts the ribosomal complex and prevents the incorporation of puromycin (Fig. 9*D*,*E*,*G*). A similar trend was also observed in presynapses (Fig. 9*C*,*F*), albeit the local translation levels were too low for a clear differentiation between RBM3 knockdowns and controls (Fig. 9*H*). Importantly, when we analyzed the effects of the knockdown at the level of the whole cells, no significant difference could be measured (Fig. 9*I*). This suggests that RBM3 controls local translation in postsynapses, and possibly also in presynapses, but its effects do not extend, under these conditions, to the organization of translation in the entire cell.

Importantly, to study the influence of molecular clock on the primary hippocampal culture, we repeated several of the assays presented above with a knockdown for a core clock gene, Brain, and muscle ARNT like 1 (BMAL1). The significant reduction in BMAL1 gene expression on BMAL1 shRNA was verified using qPCR. Surprisingly, we have not observed any substantial changes in the BMAL1 knocked down neurons (Fig. 10). This confirms that the observations we made above are specific for RBM3, which again underlines the importance of this protein for neuronal activity in these cultures.

## Discussion

Our results suggest that hippocampal neurons in dissociated cultures maintain synchronized activity patterns, which are reproducible between independent coverslips and preparations, as demonstrated by both calcium imaging and measurements of synaptic vesicle dynamics. At the same time, their transcriptomes also show a tendency to synchronize, albeit only one protein, RBM3, showed significant differences between different time points, when multiple cultures were considered. RBM3 manipulations resulted in profound changes in the neuronal activity patterns. A potential mechanism for the RBM3 function may be through its modulation of local translation in synapses (Fig. 7), which it appears to affect in a specific fashion, with less influence on global translation.

An important issue is why peaks of activity appear at similar time points across different cultures. We speculate that the time of making the cultures is relevant, and we kept this parameter constant throughout all experiments, with the rats being delivered at 10:00 A.M. to 11:00 A.M., and the resulting cultures placed in the incubator between 4:00 P.M. and 5:00 P.M. The density of the cultures may also influence their synchronization in terms of activity, as mentioned in the Introduction, and as detailed in the following paragraphs.

### Activity patterns in dissociated hippocampal neurons

Primary hippocampal cultures are prepared from mechanically and enzymatically dissociated hippocampi. The loss of the third dimension is a dramatic change for the network dynamics. At the same time, not having hormonal and temporal input from other regions makes it more difficult to synchronize the neurons in a culture. This is already known from SCN cultures, where the neurons demonstrate individual rhythmicity (Welsh et al., 1995), and can maintain 24 h rhythmicity when plated at high densities (Honma et al., 1998), but lose rhythmicity when the network communication is perturbed (Yamaguchi et al., 2003). Together, these findings suggest that network communication is essential for rhythmicity and synchronization in cultures, and that low-density cultures will lose synchronization relatively rapidly.

In view of these arguments, it was unclear whether hippocampal neurons would be able to synchronize over long periods in the culture, as the SCN neurons do (Watanabe et al., 1993). Interestingly, our findings are consistent with the observations on SCN. Dissociated hippocampal neurons exhibit high or low activity at specific times of day/night, albeit a clear 24 h rhythmicity cannot be observed when averaging results across different cultures.

Neuronal activity was not the only factor that presented such a behavior. Presynaptic activity, synapse size, and mRNA amounts at the synapse also were changing throughout 24 h. These observations suggest that one of the most commonly used models for synaptic research, the primary hippocampal culture, has a time-dependent behavior. This makes it extremely important to acknowledge the timing of experiments performed with these cultures.

### RBM3 connects molecular clock genes to neuronal function

It has been repeatedly demonstrated that the molecular clock regulates genes that control neuronal activity, as discussed in the Introduction. This makes them excellent candidates for the regulation of rhythmic activity in cultured neurons. Surprisingly, we did not find any of the core clock genes to have a very clear transcription pattern in these cultures, which implies that they may not be very well synchronized among different neurons and different cultures, unlike RMB3. This molecule has been found in many time-series transcriptomics datasets as a daily (24 h) rhythmic gene (Yan et al., 2008; Zhang et al., 2014; Pembroke et al., 2015; Renaud et al., 2015; Terajima et al., 2017; Noya et al., 2019; Ray et al., 2020), and its oscillations in expression may be independent of at least some components of the molecular clock, as they still persist in BMAL1 KO cells (Ray et al., 2020). Overall, our work cannot state whether the RBM3 oscillations are controlled by the central molecular clock machinery in hippocampal cultures. However, its stronger synchronization (across cultures) than that of the canonical clock genes implies that Rbm3 expression may be independent from them.

Other than being a rhythmically expressed gene in the literature, RBM3 is a cold-shock protein, whose expression is induced in hypothermia conditions. For example, keeping a culture at 32°C instead of 37°C for 24 h induces Rbm3 expression (Chappell et al., 2001; Yang et al., 2019). Such temperature changes are not possible in the wells of a closed plate in the incubator, which eliminates the possibility that Rbm3 expression was synchronized by temperature changes in our experiments.

As a cold shock protein, RBM3 activates the translation machinery. Several studies have demonstrated that RBM3 enhances polysome formation, by phosphorylation of translation initiation factors and by changing the miRNA level (Chappell et al., 2001; Dresios et al., 2005). RBM3 has been described to enhance the translation of specific genes in hypothermia, thereby protecting synaptogenesis (Yan et al., 2019; Zhu et al., 2019). Moreover, although RBM3 is primarily located at the nucleus, one isoform has been found in dendrites, where it colocalizes with a ribosomal protein (Smart et al., 2007). In summary, these observations suggest that RBM3 is important for synaptic function, probably because of its role in translation, and possibly in local synaptic translation. Our findings suggest that RBM3 does not change the overall mRNA availability in synapses, but that it specifically changes local translation in synapses, without affecting the global translation. This effect may result in strong changes in synaptic activity, as explained below.

### RBM3 may regulate synaptic function through local translation

Local translation appears to be an essential resource for neurons, since they need to strengthen or prune their connections in response to changes in synaptic activity. This implies that new proteins, as synaptic receptors, need to be incorporated dynamically in synapses. As neurons have extremely long neurites (Ishizuka et al., 1995), transport from the cell body would probably fail to satisfy the protein turnover needs of the synapses. To cope with this logistics challenge, neurons would need to place the translation machinery in synapses.

For a long time, electron microscopy images of synapse have demonstrated the presence of polyribosomes in the dendritic shaft and in the postsynapse (Steward and Levy, 1982; Ostroff et al., 2018). Later studies have shown that other components of the translational machinery, such as tRNAs, translation initiation factors, and elongation factors are present in synapses (Steward and Levy, 1982; Tiedge and Brosius, 1996; Sutton and Schuman, 2006). Despite these observations, direct evidence for translation in all synaptic compartments, and especially in the presynapse, has been difficult to obtain until recent assays demonstrated this thoroughly for both synaptic boutons and dendritic spines (Hafner et al., 2019).

Functional data have also offered strong support to the idea that local translation is an important feature of the synapses. Synaptic plasticity has been shown to depend on local translation (Miller et al., 2002). This process has also been linked to memory formation (Jones et al., 2018). Furthermore, electrical activity can be affected by the local translation as well, as in the case of the calyx of Held (Scarnati et al., 2018), where the inhibition of protein synthesis enhances spontaneous activity.

Overall, these observations suggest that local translation has important effects on synaptic transmission, and hence on plasticity, which last for hours. It is therefore evident that disturbing local translation would affect synaptic transmission, which in turn would influence the general network activity, as we observed in RBM3 KD experiments.

To our knowledge, this is the first time that RBM3 has been linked to changes in local translation, or to long-term neuronal activity changes. At the same time, our work demonstrates that broad changes take place in neuronal activity depending on the time of day/night, even in a simple model like dissociated hippocampal neurons in culture. This suggests that these cultures, which are far more common than SCN cultures, could become a useful model for circadian rhythm studies. Finally, the link between RBM3 and local translation may provide substantial further insight in the future, especially as the local translation field is now rapidly progressing through numerous innovative tools and concepts (Holt et al., 2019).
